# Assessing the sensitivity and suitability of a range of detectors for SIMT PSQA

**DOI:** 10.1002/acm2.14343

**Published:** 2024-04-03

**Authors:** Leon Dunn, Alessia Tamborriello, Brindha Subramanian, Xiaolei Xu, Tyrone Te Ruruku

**Affiliations:** ^1^ GenesisCare Berwick St John of God Berwick Specialist Centre Berwick Victoria Australia; ^2^ GenesisCare Frankston Frankston Public Surgical Centre Frankston Victoria Australia; ^3^ GenesisCare Ringwood Ringwood Private Hospital Ringwood East Victoria Australia

**Keywords:** ArcCHECK, detector, external beam, film, log file, portal dosimetry, PSQA, quality assurance, sensitivity, SIMT, SRS, SRS MapCHECK, SRT, stereotactic

## Abstract

**Purpose:**

Single‐isocenter multi‐target intracranial stereotactic radiotherapy (SIMT) is an effective treatment for brain metastases with complex treatment plans and delivery optimization necessitating rigorous quality assurance. This work aims to assess five methods for quality assurance of SIMT treatment plans in terms of their suitability and sensitivity to delivery errors.

**Methods:**

Sun Nuclear ArcCHECK and SRS MapCHECK, GafChromic EBT Radiochromic Film, machine log files, and Varian Portal Dosimetry were all used to measure 15 variations of a single SIMT plan. Variations of the original plan were created with Python. They comprised various degrees of systematic MLC offsets per leaf up to 2 mm, random per‐leaf variations with differing minimum and maximum magnitudes, simulated collimator, and dose miscalibrations (MU scaling). The erroneous plans were re‐imported into Eclipse and plan‐quality degradation was assessed by comparing each plan variation to the original clinical plan in terms of the percentage of clinical goals passing relative to the original plan. Each erroneous plan could be then ranked by the plan‐quality degradation percentage following recalculation in the TPS so that the effects of each variation could be correlated with γ pass rates and detector suitability.

**Results & conclusions:**

It was found that 2%/1 mm is a good starting point for the ArcCHECK, Portal Dosimetry, and the SRS MapCHECK methods, respectively, and provides clinically relevant error detection sensitivity. Looser dose criteria of 5%/1 mm or 5%/1.5 mm are suitable for film dosimetry and log‐file‐based methods. The statistical methods explored can be expanded to other areas of patient‐specific QA and detector assessment.

## 1 INTRODUCTION

The clinical management of patients with brain metastases has changed in recent years, with a shift away from whole‐brain radiation therapy (WBRT) to fractionated stereotactic radiosurgery (SRS). Single‐isocenter multi‐target radiotherapy (SIMT) delivered in one to five fractions has been shown to offer excellent local control while maintaining acceptable toxicity in the treatment of multiple intact brain metastases.^1^, ^2^

Stereotactic radiotherapy treatment has become the recommended treatment for patients with one to four metastases, demonstrating equivalent survival and a lower risk of long‐term neurocognitive decline, compared with SRS plus WBRT.^3^, ^4^, ^5^ Further, similar survival and preservation of neurocognitive function have been shown in patients receiving SRS SIMT for more than five metastases.^6^, ^7^, ^8^ This modern treatment option is more efficient and less invasive compared with historical options of WBRT, surgery, radiosurgery, radiosensitizers, and chemotherapy.

The SIMT technique uses automated treatment planning techniques to optimize target coverage and organ‐at‐risk (OAR) sparing. Two modes of delivery currently available for SIMT plans are the Varian Hyperarc (HA) technique (Varian Medical Systems, Palo Alto, California, USA),^9^, ^10^ which utilizes volumetric modulated arc therapy (VMAT), and the BrainLab Elements Multiple Brain Metastases software (BrainLab, Munich, Germany), which utilizes dynamic conformal arc therapy (DCAT).^11^

SIMT plans are optimized to deliver large and highly conformal dose distributions to multiple small volumes utilizing an idealized treatment system in terms of imaging, localization, and delivery. While SIMT offers excellent local control and acceptable toxicity, it is less clear how sensitive these plans are to sub‐optimal machine performance and geometric localization variations. What is displayed to the dosimetrist or clinician in terms of target coverage and acceptable OAR toxicity may not be achievable due to localization and machine performance uncertainties. It is therefore pertinent to conduct robust patient‐specific quality assurance (PSQA) measurements for all SIMT plans on the intended treatment machine to assess the deliverability, dosimetry, and localization of the dose deposition.

A range of quality assurance (QA) tools are reported for use in the QA of stereotactic ablative body radiotherapy (SABR) and SIMT treatment plans. Some commonly available methods and sample publications are listed below:

- EBT3 and EBT‐XD Radiochromic Film^12^ (Ashland Specialty Products, Wilmington, Delaware, USA)
- Low and high detector‐density ion chamber/diode arrays:
  ⚬ SRS MapCHECK,^13^, ^14^ ArcCHECK,^15^ and MapCHECK 2^16^ (Sun Nuclear Corporation, Melbourne, Florida, USA).
  ⚬ PTW Octavius, Octavius II, Octavius Detector 1600 SRS^17^ PTW Freiburg GmbH, Freiburg, Germany)
  ⚬ IBA myQA SRS Detector (IBA International, Louvain‐La‐Neuve, Belgium)
- Small‐volume ion chambers/diamond detectors (point dose measurements)
- Electronic Portal Imaging Device (EPID) based 2D or 3D back projection reconstruction techniques:
  ⚬ Varían Portal Dosimetry (PD) (Varían Medical Systems, Palo Alto, California, USA)
  ⚬ Sun Nuclear 3DVH, PerFRACTION
  ⚬ VIPER^18^ Calvary Mater Newcastle Hospital, New South Wales, Australia)
- Polymer Gel dosimetry^19^
- Machine delivery log files and independent recalculation (Mobius 3D, Varian Medical Systems, Palo Alto, California, USA)^20^, ^21^

The tools listed above each have pros and cons, as well as cases for where they are best suited. In this study, we compare EBTXD Radiochromic Film, the SNC ArcCHECK and SRS MapCHECK, Varian Portal Dosimetry, and TrueBeam log file (trajectory log) analysis to determine each detector/method's ability to detect clinically significant errors in the context of SIMT. We propose that a suitable detector should, at a minimum, be able to detect any deviation in machine performance and/or error induced in the plan that can be shown to have a clinical impact on the plan quality. We hypothesize that appropriate gamma (γ) criteria can be chosen irrespective of the detector, by testing for a criterion set (dose difference/distance‐to‐agreement) that decreases the γ‐passing rate relative to the ground truth linearly in proportion with the severity of the effect.

Using this methodology, we compare EBT‐XD Radiochromic Film, the SNC ArcCHECK and SRS MapCHECK, Varian PD, and TrueBeam log file (trajectory log) analysis to determine each detector/method's ability to detect clinically significant errors in the context of SIMT. While this work is presented in the context of SIMT, this method is extendable to other techniques and detectors (IMRT, VMAT, SABR, etc.) and is a novel way to determine the optimal γ criteria to use for these devices/methods.

## 2 MATERIALS AND METHODS

### 2.1 Materials: Clinical case

**FIGURE 1:**
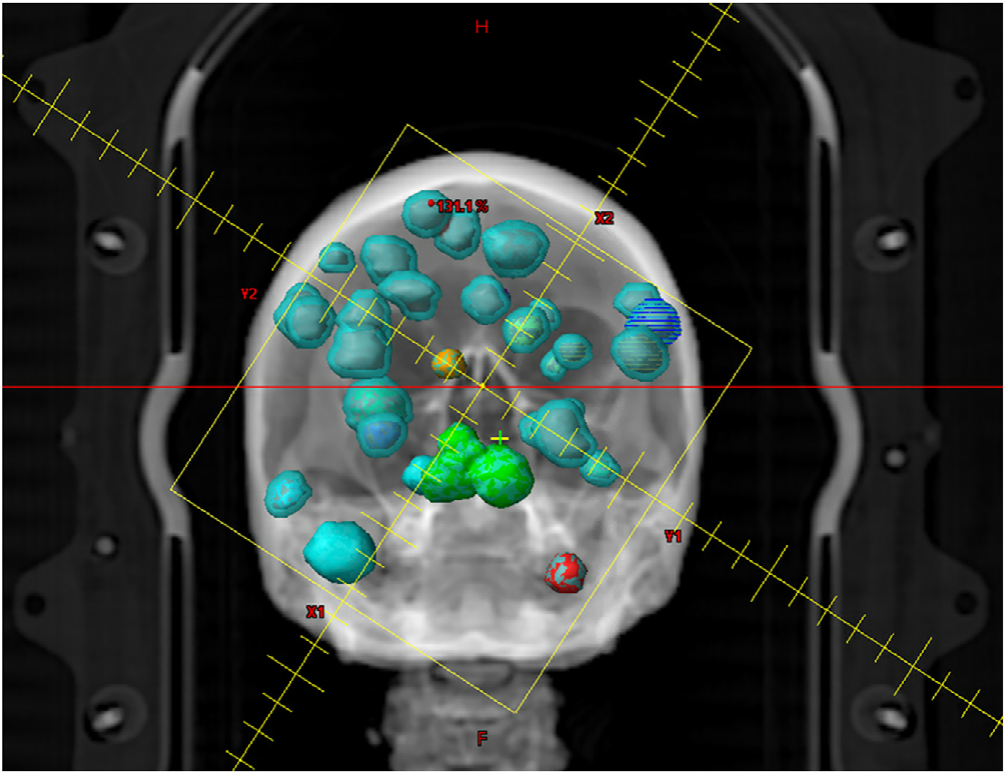
3D Visualization of the treatment case in Eclipse v16.1. As the distribution was throughout the brain, the plan had 180° arcs at couch angles of 0°, 315°, 45°, and 90° respectively. The plan was created without jaw tracking to enable introducing errors in the plan as discussed in Section 2.3.

A single patient with multiple brain metastases treated with stereotactic radiotherapy using the HyperArc technique was chosen for this retrospective study based on the complexity of the case, and the size and distribution of the 21 individual planning target volumes (PTVs) ranging from 0.4 to 8.1 cc. A 3D rendering of the case is shown in Figure 1. The patient had previously undergone stereotactic radiotherapy as well as WBRT. The dose calculation algorithm used was the Eclipse AcurosXB algorithm (v16.1) with 0.125 cm calculation resolution reporting dose to medium. The plan was originally measured with EBT‐XD Radiochromic film and treated on a Varian TrueBeam Edge linear accelerator with a High‐Definition Multi‐Leaf Collimator (HDMLC) (Varian Medical Systems, Palo Alto, California, USA) and AlignRT surface guidance (VisionRT Ltd, London, UK, N3 2JU).

### 2.2 Materials: Detectors and associated equipment

The following tools were used in this study:

- Sun Nuclear ArcCHECK
- Varian PD
- Radiochromic film (EBT3/EBT‐XD)
- Varian TrueBeam log files
- Sun Nuclear SRS MapCHECK
- CIRS Multi‐Lesion Brain QA Phantom Model 037
- “Luki‐Phan” a 3D‐printed phantom for use with the SRS MapCHECK

The detectors used, their features, and acquisition class according to AAPM TG‐218 are shown in the Appendix (Table A1). Radiochromic film was used in conjunction with the CIRS Multi‐Lesion Brain QA phantom (Model 037), and the SRS MapCHECK was used in conjunction with the Luki Phan, which is an in‐house 3D‐printed dice‐shaped phantom. The Varian Portal Dosimetry method is EPID‐based and does not require a phantom. Equally, Varian TrueBeam trajectory log files also require no phantom or phantom measurement. The detectors have varying levels of comprehensiveness to which they measure the absolute dose and dose distribution for the gantry, collimator, couch angles, and field size as per the plan and in simulated patient geometry. A summary of the detectors used is provided in the Appendix (Table A1).

### 2.3 Methods: Python scripting to introduce errors

To generate the erroneous plans with simulated MLC errors, the original plan DICOM file was exported, anonymized, and then modified using a Python script (Version 2.7). The script imports the DICOM file using the Pydicom module (https://pydicom.github.io/) and for each control point in the beam sequence a modification is performed on all the MLC leaves. The modified plan is then saved and can be re‐imported into the Eclipse Treatment Planning System (TPS) for comparison. For this method to work, a copy of the original plan without jaw‐tracking needed to be created and it was this plan that was modified. The copy of the original plan without jaw‐tracking is the ground truth in this work. The list of modifications is shown in Table 1.

**TABLE 1:** List of erroneous plans generated.

Plan/Rank #	Modification	Percentage of clinical goals passing	PTV_TOTAL D98%	PTV_TOTAL D2%	PTV_TOTAL Dmean [%]
Brain 0	Original plan	97.5	99.2	126.3	112.0
Brain 1	1% scaling of MU	95.8	100.1	127.6	113.1
Brain 2	2% scaling of MU	92.5	101.2	128.9	114.3
Brain 3	3% scaling of MU	91.7	102.1	130.1	115.4
Brain 4	0.01–0.1 mm random offset	91.7	99.1	126.3	112.1
Brain 5	0.1 mm systematic	90.8	98.9	126.4	112.1
Brain 6	0.1–0.25 mm random	85.8	98.2	126.4	112.0
Brain 7	7% scaling of MU	85.0	106.1	119.9	135.2
Brain 8	1‐degree collimator rotation	85.0	98.0	126.5	111.9
Brain 9	0.25 mm systematic offset	82.5	96.7	126.4	111.7
Brain 10	0.25–0.5 mm random offset	81.7	95.6	126.4	111.5
Brain 11	2‐degree collimator rotation	74.2	94.9	126.5	111.0
Brain 12	0.1 – 1 mm random offset	63.3	87.5	126.2	109.3
Brain 13	0.5 mm systematic offset	60.0	87.8	108.0	108.0
Brain 14	1 mm systematic offset	40.0	70.0	125.2	102.8
Brain 15	1–2 mm random offset	29.2	53.4	121.8	93.1

The list is sorted against the percentage of clinical goals passing compared to the original plan, “Brain 0.” The decrease in the percentage of clinical goals passing is an indicator of the degradation of plan quality and therefore a measure of the severity of the error. Where modifications refer to “systematic” or “random” offsets, this refers to all MLC leaf positions per control point. Where collimator rotations are mentioned, this refers to a collimator rotation angle increase by the degree amount to all fields in the original plan.

### 2.4 Methods: Assessing the impact on plan quality

**FIGURE 2:**
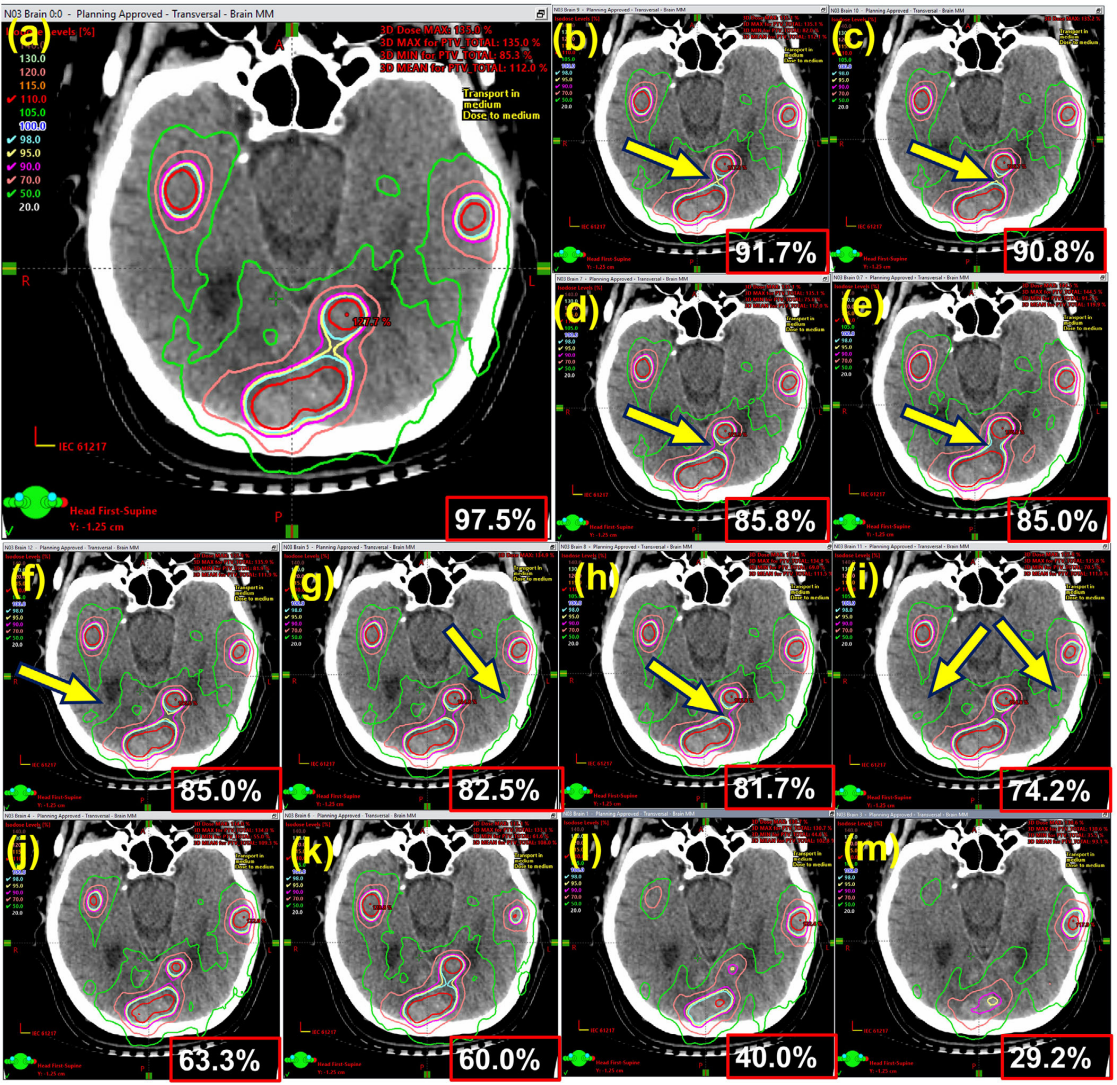
Mosaic showing the degree of plan quality degradation compared to the original plan (a), when the errors from Table 1 are introduced to the plan files and re‐imported into Eclipse. (b) shows the dose distribution of a single slice with 0.01–0.1 mm random offsets applied to each MLC leaf per control point. The arrows point to features of the isodose distribution that change relative to the original plan. (c)—(m) show continuous degradation with larger errors introduced from cases: Brain 6−10 and Brain 13−16, respectively. The percentage of clinical goals passing is shown in the bottom right box in each image.

To determine the plan quality impact and therefore potential clinical impact, the error‐laden plans generated in Section 2.3 were re‐imported into the TPS for recalculation. The clinical severity of an error was then determined by ranking the percentage of clinical goals passing relative to the original clinical plan. For example, scaling the monitor units by 1% in an artificially modified plan decreases the percentage of clinical goals passing from 97.5% (original plan) to 95.8% (modified plan), which has an effect, but is small relative to randomly shifting all the MLC leaves by an amount between 0.25 and 0.5 mm, which results in a reduction of 15.8% (97.5–% to 81.7%) to the percentage of clinical goals passing. Using this method, the most appropriate γ criteria for each detector were determined by the best linear model fit to the measurement result as a function of the severity of the error (as determined by the decrease in the percentage of clinical goals passing). Figure 2 shows a mosaic example of the changing isodose structures because of these introduced errors.

### 2.5 Methods: Measurements

All measurements were carried out on three dosimetrically matched TrueBeam linear accelerators that satisfy AAPM TG‐142 stereotactic performance requirements. Film measurements were repeated on two linear accelerators (Linac 1 and Linac 2), the ArcCHECK and MapCHECK were carried out on a single accelerator (Linac 1), and the Portal Dosimetry measurements were on the third accelerator (Linac 3). All three TrueBeams are equipped with Millenium MLC systems and PerfectPitch 6 degrees‐of‐freedom couches (6DOF). Periodic QA (daily/monthly/annual) is performed on all machines. Verification plans were created for the original plans for analysis with ArcCHEK, MapCHECK, and film phantom datasets. Analysis for each detector was done in absolute dose mode with a dose threshold of 10% and γ criteria of 1%/1 mm, 2%/2 mm, 3%/3 mm, 5%/1.5 mm, and 5%/1 mm, respectively.

#### 2.5.1 ArcCHECK

**FIGURE 3:**
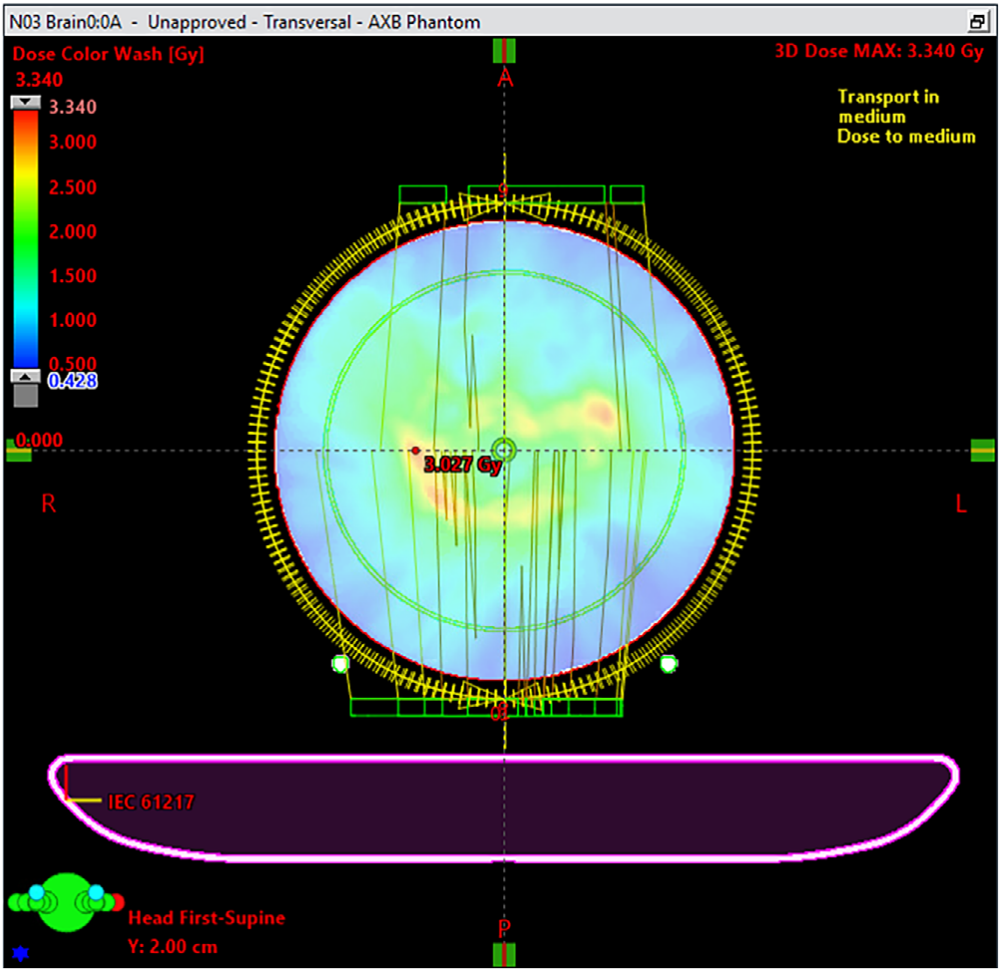
Eclipse screenshot of the plan transferred to the ArcCHECK.

The ArcCHECK (Figure 3) was set up on the TrueBeam PerfectPitch treatment couch and positioned at the isocenter using the lasers and alignment markings on the detector cylinder. Orthogonal (anterior and lateral) MV pair fields were used to verify the setup by comparison to the TPS. The standard ArcCHECK Dose Calibration procedure was then performed. All fields for the plans in Table 1 were delivered and integrated for the total dose distribution to be compared with the TPS using SNCPatient software (Sun Nuclear Corporation, Melbourne, Florida, USA). No “calc shift” registration between the delivered and planned dose distributions was performed.

#### 2.5.2 Varian portal dosimetry

**FIGURE 4:**
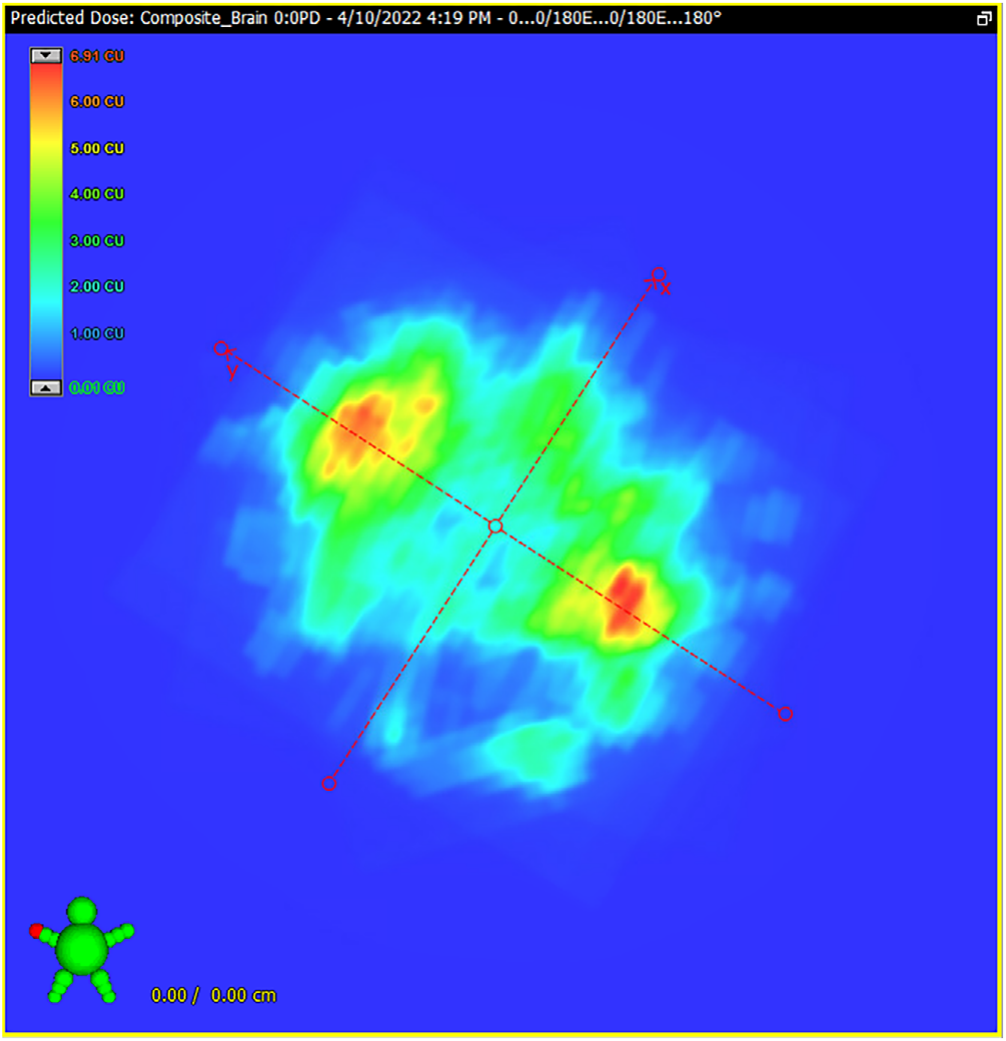
PD predicted image for the composite dose distribution of the four fields.

For each plan in Table 1, a PD Verification Plan was created in Eclipse (Figure 4). Each plan was then delivered to the EPID (positioned at 0,0,0 cm) field‐by‐field using integrated MV images. The PD software was then used to create the composite from the individual fields for comparison to the TPS‐predicted image.

#### 2.5.3 Radiochromic film

**FIGURE 5:**
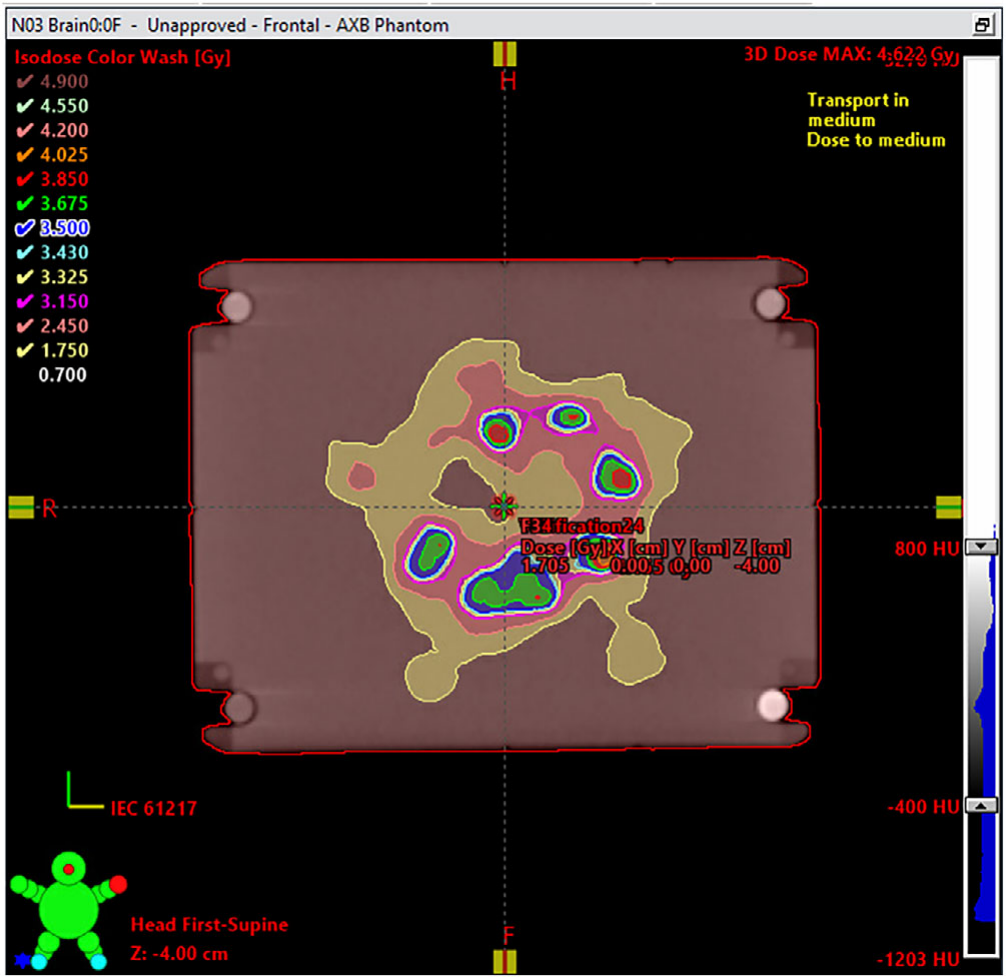
CIRS Multi‐Lesion Brain QA phantom at −4.0 cm (anterior of isocenter) showing the location of the measurement slice and the isodose distribution. Six lesions can be seen.

EBT‐XD in the CIRS Multi‐Lesion Brain QA Phantom Model 037 (Figure 5) was aligned to the lasers with a single piece of film at the central 0.0 cm slice. Registration points were marked at known distances for registration and aligned to the lasers, the phantom was reassembled, and a single plan was delivered. This setup was used for all plans, which resulted in 16 film measurements. In routine clinical practice, a film placed at each slice intersecting a PTV in the verification plan is standard. However, managing 22 PTVs and 16 plans this way would demand 352 film measurements, making it impractical for this study. Therefore, because an error‐laden plan affects all PTVs in some way, a single‐slice analysis was deemed sufficient to determine the overall effect. Films were digitized after 20 h on an Epson 11000XL flatbed scanner (Seiko Epson Corporation, Suwa, Nagano, Japan) creating 48‐bit color images with 72 dpi resolution.

#### 2.5.4 Varian TrueBeam log files

Machine log files produced during portal dosimetry measurements on Linac 3 were retrieved for in‐house processing and comparison. Each log file was converted to a fluence map of the differential MU per control point delivered by explicitly modeling the MLCs and their motion in MATLAB and adding the differential MU to each control point. This same method can be applied to the plan DICOM MLC positions meaning plan‐calculated fluence maps can be compared to log file‐generated maps. Further, log files contain all the information about the planned “expected” positions of all the mechanical axes, and the actual measured position (“actual”) fed back to the Linac. In this work we compared the planned (TPS, DICOM generated) fluence maps to the log file generated fluence maps (plan vs. log), and the log file expected versus actual (log file only method). The two methods produce different results due to the temporal resolution of the data.

#### 2.5.5 SRS MapCHECK

**FIGURE 6:**
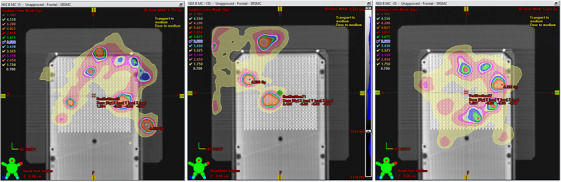
Three coronal dose plane locations and visual isodoses as depicted in Eclipse. The three planes captured 10 out of 22 PTVs. Every error‐laden plan from Table 1 was measured and compared to the TPS dose distribution as the reference.

The SRS MapCHECK was used to measure three separate coronal planes of the treatment plan capturing in total 10 out of 22 PTVs. The SRS MapCHECK was installed in the custom‐made “LukiPhan” dice‐shaped phantom and aligned to the lasers using the inscribed markings. Cone‐beam CT (CBCT) images of the phantom were used to precisely match and position the phantom in congruence with the treatment plan's reference CT. For each measurement, the four fields were delivered, and the phantom was then shifted to the next measurement plane. The reference plan and all the error introduced plans were delivered to collect data from each plane. The gamma passing rate for measurements utilizing this device was then evaluated for the captured PTVs on each individual plane for three coronal planes and the mean value of the pass rates presented. Figure 6 shows the SRS MapCHECK in Eclipse housed in the LukiPhan with three dose planes capturing multiple PTVs in each plane and the isodose lines of three separate planes overlaid.

### 2.6 Methods: Analysis methodology

For all detectors used in this study, γ analysis was performed in absolute dose mode with a threshold of 10% and dose difference/DTA criteria of 1%/1 mm, 2%/2 mm, 3%/3 mm, 5%/1.5 mm, and 5%/1 mm, respectively. To determine the ideal γ criteria for this method, a linear model of the form y = β_0_+β_1_X_1_+ϵ was used to estimate a linear fit to the measurement γ results versus the percentage of clinical goals passing, which itself is a measure of the severity of the error. For an idealized detector, the passing rate should linearly decrease with the severity of the introduced error and thus we hypothesized that an appropriate γ criteria set (dose difference/DTA) should exhibit a correlation to this parameter. The model's root mean squared error (RMSe), which estimates the standard deviation of the error distribution, the R‐squared and adjusted R‐squared coefficient of determination and adjusted coefficient of determination, respectively, and the F‐statistic versus constant model and *p* value for the F‐test on the model are reported.

- Root mean squared error—Square root of the mean squared error, which estimates the standard deviation of the error distribution. For example, a low RMSe at a given gamma criteria indicates the detector's decrease in pass rate is tightly correlated to a linear decrease in the percentage of clinical goals passing.
- R‐squared and Adjusted R‐squared are the coefficient of determination and adjusted coefficient of determination, respectively. For example, at 2%/1 mm the ArcCHECK has an *R* ^2^ = 0.92 (92%) (Table 2), which indicates that the model fits the data well. At this dose/dta criteria, the detector can detect the introduced errors which have been shown to have an impact on the number of clinical goals passing. At 3%/3 mm, the *R* ^2^ = 0.73 (73%) demonstrating that the detector fails to have gamma pass‐rates that correlate well to potential clinical impact.
- F‐statistic versus constant model—Test statistic for the F‐test on the regression model, which tests whether the model fits significantly better than a degenerate model consisting of only a constant term. The result is significant if the F statistic is larger because this indicates greater differences among the sample averages.
- *p* value—*p* value for the F‐test on the model. If the *p* value is low and the F‐statistic is large, then the overall results are significant.

**TABLE 2:** Linear model results comparing a linear fit of the decline in γ pass‐rates against plan‐quality degradation as measured by the percentage of clinical goals passing following the introduction of errors to the treatment plan.

Detector	γ Criteria	RMSe	R^2^	Adj. R^2^	F‐statistic vs. const. model	p‐value
ArcCHECK	1%/1mm	9.0	0.81	0.80	59.8	2.02 × 10^−6^
	2%/1mm	5.7	0.92	0.92	169.0	3.27 × 10^−9^
	3%/3mm	10.7	0.73	0.71	38.1	2.41 × 10^−5^
	5%/1.5mm	7.8	0.86	0.85	85.3	2.48 × 10^−7^
	5%/1mm	5.1	0.94	0.93	214.0	7.04 × 10^−10^
PD	1%/1mm	7.8	0.86	0.85	84.1	2.69 × 10^−7^
	2%/1mm	4.9	0.95	0.94	239.0	3.39 × 10^−10^
	3%/3mm	11.0	0.72	0.70	35.6	3.43 × 10^−5^
	5%/1.5mm	8.0	0.85	0.84	80.8	3.44 × 10^−7^
	5%/1mm	7.5	0.87	0.86	92.3	1.53 × 10^−7^
Film	1%/1mm	17.8	0.26	0.21	5.0	0.043
	2%/1mm	18.8	0.18	0.12	3.0	0.103
	3%/3mm	18.0	0.25	0.19	4.6	0.050
	5%/1.5mm	16.8	0.34	0.29	7.2	0.018
	5%/1mm	17.7	0.27	0.22	5.2	0.039
Log File vs. Plan	1%/1mm	8.9	0.82	0.80	62.1	1.63 × 10^−6^
	2%/1mm	7.3	0.88	0.87	99.9	9.42 × 10^−8^
	3%/3mm	8.1	0.85	0.84	77.2	4.53 × 10^−7^
	5%/1.5mm	4.6	0.95	0.95	275.0	1.34 × 10^−10^
	5%/1mm	3.7	0.97	0.97	417.0	8.09 × 10^−12^
Log File Actual vs. Log File Expected	1%/1mm	13.0	0.61	0.58	21.5	0.0004
	2%/1mm	8.1	0.85	0.84	77.9	4.28 × 10^−7^
	3%/3mm	8.5	0.83	0.82	70.2	7.95 × 10^−7^
	5%/1.5mm	4.5	0.95	0.95	278.0	1.24 × 10^−10^
	5%/1mm	3.1	0.98	0.98	606.0	6.36 × 10^−13^
SRS MapCHECK	1%/1mm	10.1	0.76	0.75	44.8	1.02 × 10^−5^
	2%/1mm	9.9	0.77	0.76	47.6	7.3 × 10^−6^
	3%/3mm	12.1	0.66	0.63	26.9	0.0001
	5%/1.5mm	9.8	0.78	0.76	49.2	6.11 × 10^−6^
	5%/1mm	9.0	0.81	0.80	59.8	2.03 × 10^−6^

The highlighted rows per detector show the favorable criteria that yield measurement results that are strongly correlated with detecting the error.

## 3 RESULTS

### 3.1 Results summary

**FIGURE 7:**
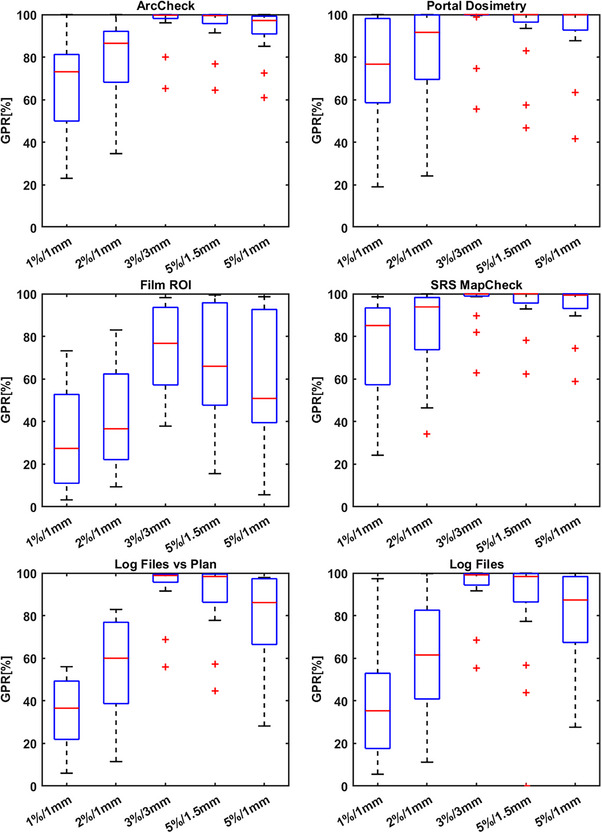
Boxplot distributions for the γ results per criteria for each detector. A larger range of GPR in this work is favorable since the GPR should decrease in proportion to the severity of the error introduced to the delivery. An ideal detector would pass for the original plan (Brain 0) and fail for all other plans (Brain 1–15) in Table 1.

**FIGURE 8:**
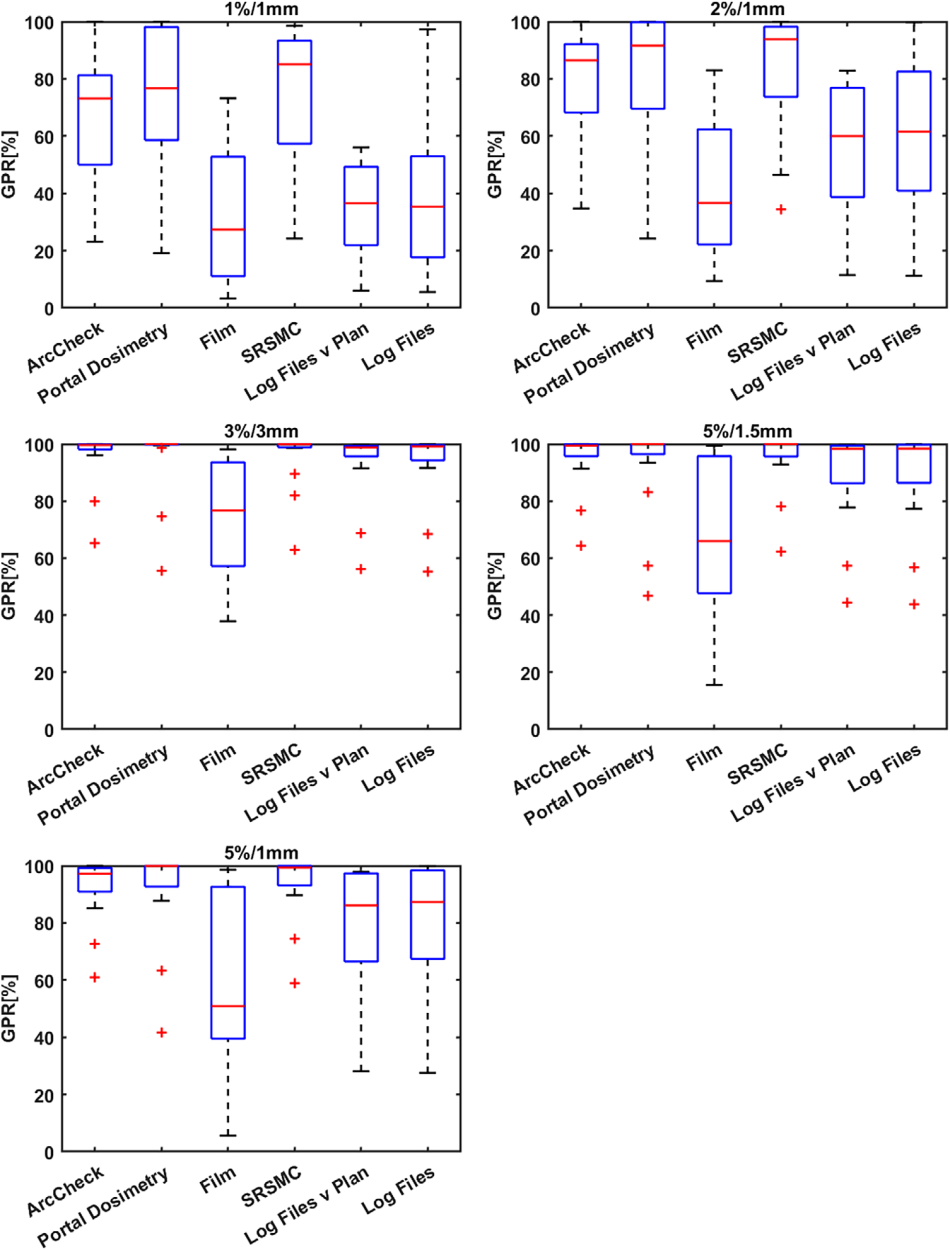
Summary of the measurement results grouped by γ‐criteria. 5%/1 mm or 2%/1 mm are the most suitable criteria across all detectors providing the best error detection.

Figure 7 presents a boxplot summary of detector results. The ideal γ criteria for each detector aim to pass the ground‐truth plan error‐free and detect errors proportionate to their severity based on γ pass rates (GPR). A wider interquartile range and overall range in these cases indicate greater error detection sensitivity. A key result of this study is that these results demonstrate that a 3%/3 mm criterion is unsuitable for all detectors except film due to additional uncertainty. For instance, PD with 3%/3 mm detects only severe errors. Error‐laden plans had a 100% pass rate, except for Brain 14 and 15, which caused substantial plan degradation due to systematic MLC shifts and randomized leaf offsets. These errors could lead to significant mistreatment, reducing clinical goal achievement to 40.0% and 29.7%, respectively, from an initial 97.5%. Figure 8 shows a summary of the detector results grouped by γ criteria. Figure 8 presents the same results grouped by γ criteria.

The results of the linear model are shown in Table 2. The linear model was a fit of the results to the ranked decline of clinical goals passing (see Table 1). Table 2 shows the parameters of the model for each detector and γ‐criteria. The best fit of the model was used to determine the optimal γ‐criteria which is shaded in the table. According to this model, the optimal criteria for ArcCHECK were 2%/1 mm and 5%/1 mm, respectively. For the PD method, 2%/1 mm should be used to provide the best error detection. For film, 5%/1.5 or 5%/1 mm should be used. For log files, which compare reconstructed fluence maps from MLC positions and differential control point MU integration, 2%/1 mm or 5%/1 mm provide the best error detection. Finally, for the SRS MapCHECK, 2%/1 mm provided the best error detection in this study.

### 3.2 ArcCHECK

**FIGURE 9:**
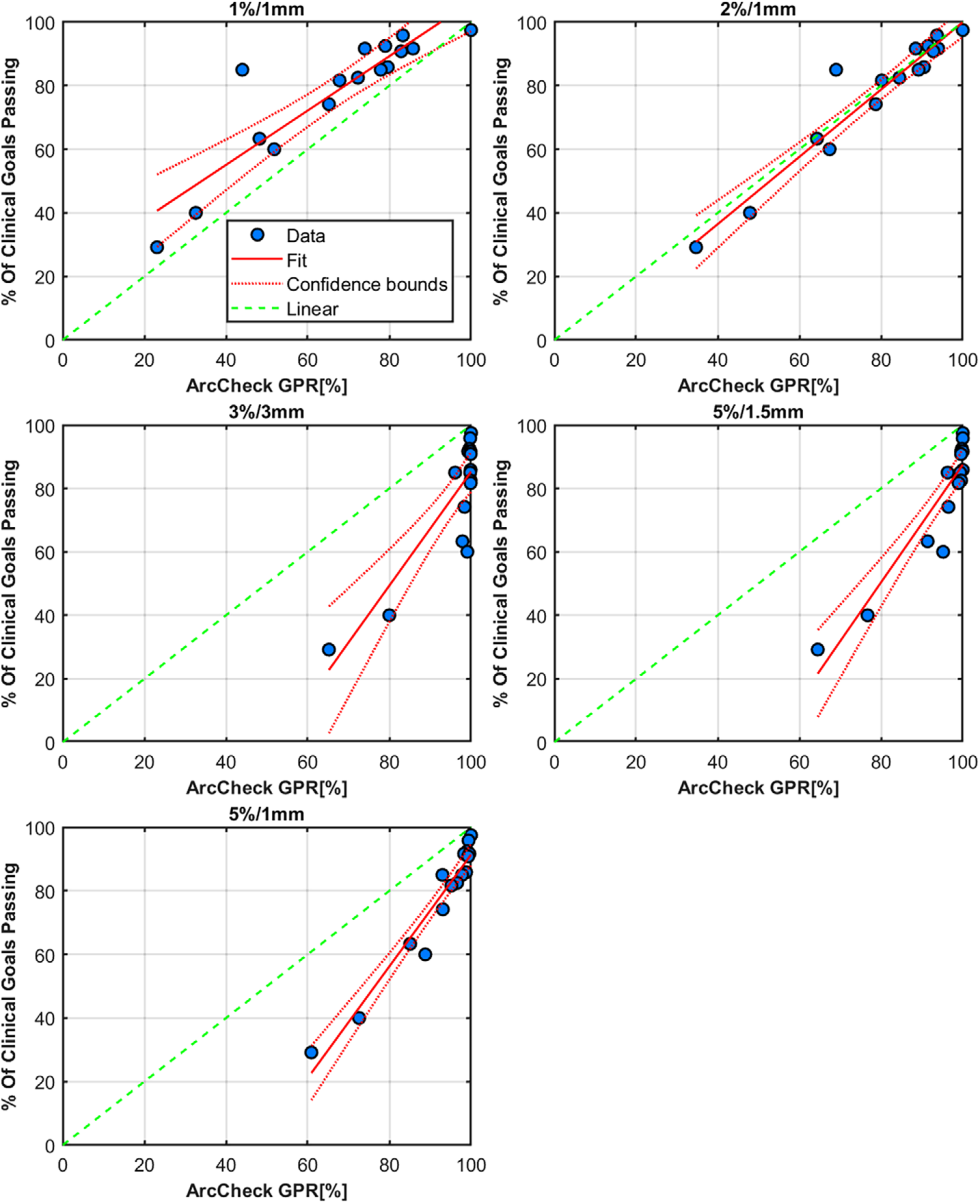
ArcCHECK results for 1%/1 mm (top), 2%/1 mm, 3%/3 mm, 5%/1.5 mm, and 5%/1 mm (bottom) respectively. Each measurement point corresponds to a planned delivery from Table 1. The measurement points are represented by circles, with a linear model fit to the data along with confidence intervals for the model shown in a solid and dashed red line, respectively. The green dashed line indicates ideal linearity.

Results for the ArcCHECK measurements and the linear modeling are shown in Figure 9. The original plan passed at 100% for all γ‐criteria evaluated. The most appropriate criteria were found via the linear model to be 5%/1 mm and 2%/1 mm, respectively. Table 3 shows the results for all ArcCHECK plans measured for criteria of 1%/1 mm, 2%/2 mm, 3%/3 mm, 5%/1.5 mm, and 5%/1 mm, respectively.

**TABLE 3:** ArcCHECK GPR results for all plans measured.

Plan	Modification	1%/1 mm [%]	2%/1 mm [%]	3%/3 mm [%]	5%/1.5 mm [%]	5%/1 mm [%]	Clinical goals passing [%]
Brain 0	Original Plan	100.0	100.0	100.0	100.0	100.0	97.5
Brain 1	1% scaling of MU	83.3	93.7	99.8	100.0	99.4	95.8
Brain 2	2% scaling of MU	79.0	91.5	99.6	99.8	99.1	92.5
Brain 3	3% scaling of MU	74.0	88.5	99.3	99.6	98.3	91.7
Brain 4	0.01–0.1 mm random offset	85.8	94.0	99.9	100.0	99.6	91.7
Brain 5	0.1 mm systematic	82.9	92.8	99.9	99.6	99.3	90.8
Brain 6	0.1‐0.25 mm random	79.6	90.5	99.9	100.0	98.8	85.8
Brain 7	7% scaling of MU	44.0	69.0	96.1	96.3	93.0	85.0
Brain 8	1‐degree collimator rotation	77.9	89.2	99.8	99.4	97.8	85.0
Brain 9	0.25 mm systematic offset	72.3	84.5	99.9	99.6	96.6	82.5
Brain 10	0.25–0.5 mm random offset	67.8	80.2	99.9	99.0	95.2	81.7
Brain 11	2‐degree collimator rotation	65.2	78.7	98.4	96.5	93.1	74.2
Brain 12	0.1–1 mm random offset	48.2	64.3	97.9	91.4	85.1	63.3
Brain 13	0.5 mm systematic offset	51.8	67.4	99.1	95.2	88.8	60.0
Brain 14	1 mm systematic offset	32.6	47.9	80.0	76.7	72.6	40.0
Brain 15	1–2 mm random offset	23.1	34.7	65.2	64.5	60.9	29.2

Shaded regions show optimal criteria as determined by the linear model.

### 3.3 Portal dosimetry

**FIGURE 10:**
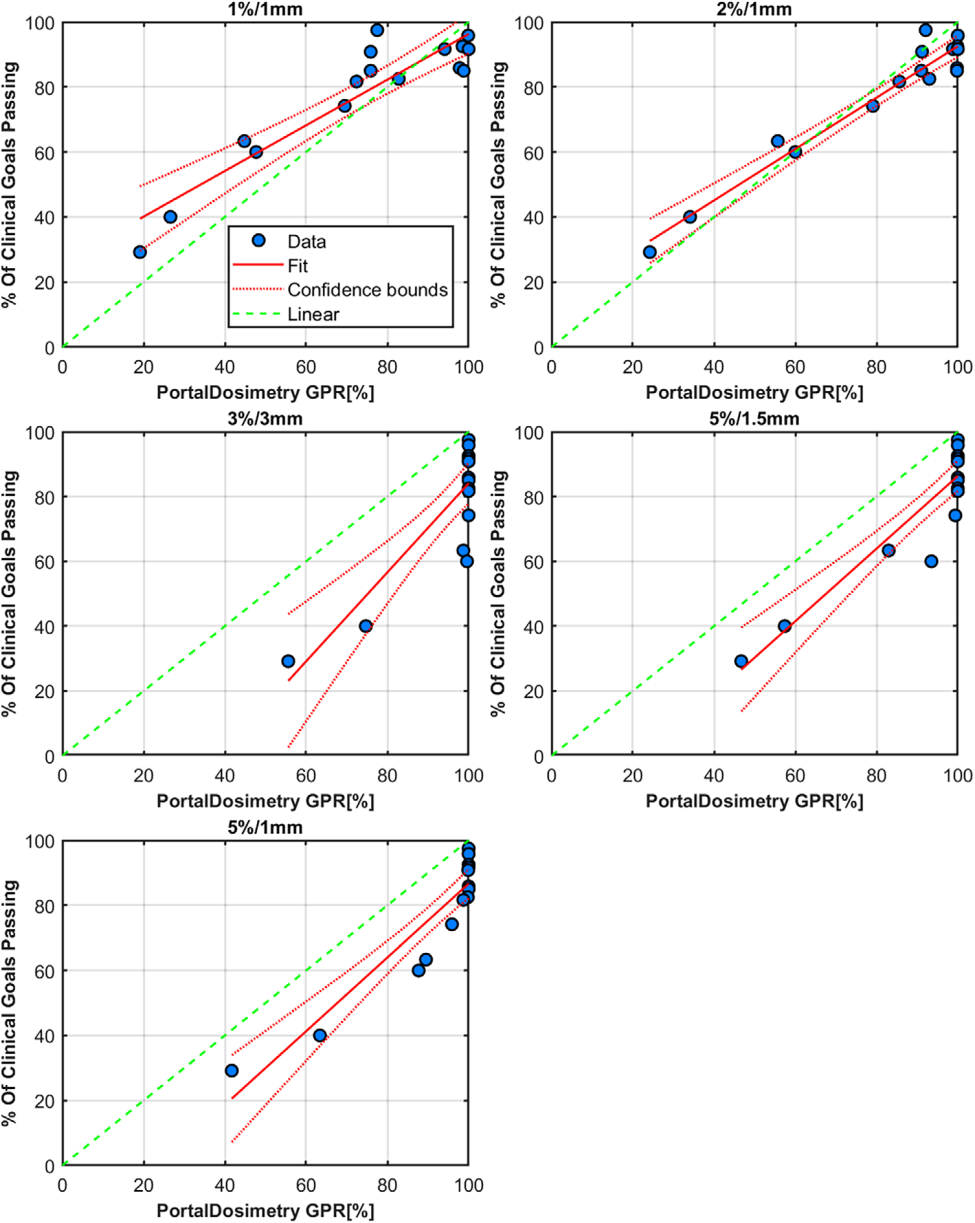
Results from Varian's PD software. Each figure's title shows the γ criteria used for the analysis. Each data point is a comparison of the composite dose from four fields analyzed in PD for the plans in Table 1. Each measurement of the original and erroneous plan is compared to the original plan's portal dose image prediction composite.

Results for the PD measurements and the linear modeling are shown in Figure 10. The original plan passed at 100% for all γ criteria except for 2%/1 mm and 1%/1 mm. The most appropriate criteria were found via the linear model to be 2%/1 mm, even though this criterion reported a failed result for the original plan. The range of γ pass rates for this criterion was 68%. At 2%/1 mm all plans with errors introduced failed except for Brain 1, where the scaling of the MU by 1% improved the result. Table 4 shows the results for all PD plans measured for criteria of 1%/1 mm, 2%/2 mm, 3%/3 mm, 5%/1.5 mm, and 5%/1 mm, respectively.

**TABLE 4:** PD GPR results for all plans measured.

Plan	Modification	1%/1 mm [%]	2%/1 mm [%]	3%/3 mm [%]	5%/1.5 mm [%]	5%/1 mm [%]	Clinical goals passing [%]
Brain 0	Original plan	77.5	92.1	100.0	100.0	100.0	97.5
Brain 1	1% scaling of MU	99.9	100.0	100.0	100.0	100.0	95.8
Brain 2	2% scaling of MU	98.5	99.9	100.0	100.0	100.0	92.5
Brain 3	3% scaling of MU	94.1	98.8	100.0	100.0	100.0	91.7
Brain 4	0.01–0.1 mm random offset	100.0	100.0	100.0	100.0	100.0	91.7
Brain 5	0.1 mm systematic	75.9	91.2	100.0	100.0	99.9	90.8
Brain 6	0.1–0.25 mm random	97.8	99.8	100.0	100.0	100.0	85.8
Brain 7	7% scaling of MU	75.9	91.0	100.0	100.0	100.0	85.0
Brain 8	1‐degree collimator rotation	98.8	99.8	100.0	100.0	100.0	85.0
Brain 9	0.25 mm systematic offset	82.8	93.0	100.0	100.0	99.8	82.5
Brain 10	0.25–0.5 mm random offset	72.4	85.6	100.0	100.0	98.7	81.7
Brain 11	2‐degree collimator rotation	69.5	79.1	100.0	99.4	95.9	74.2
Brain 12	0.1–1 mm random offset	44.8	55.7	98.7	83.0	89.5	63.3
Brain 13	0.5 mm systematic offset	47.7	60.0	99.6	93.5	87.7	60.0
Brain 14	1 mm systematic offset	26.6	34.1	74.7	57.4	63.4	40.0
Brain 15	1–2 mm random offset	19.1	24.2	55.6	46.7	41.7	29.2

Shaded regions show optimal criteria as determined by the linear model.

### 3.4 Radiochromic film

**FIGURE 11:**
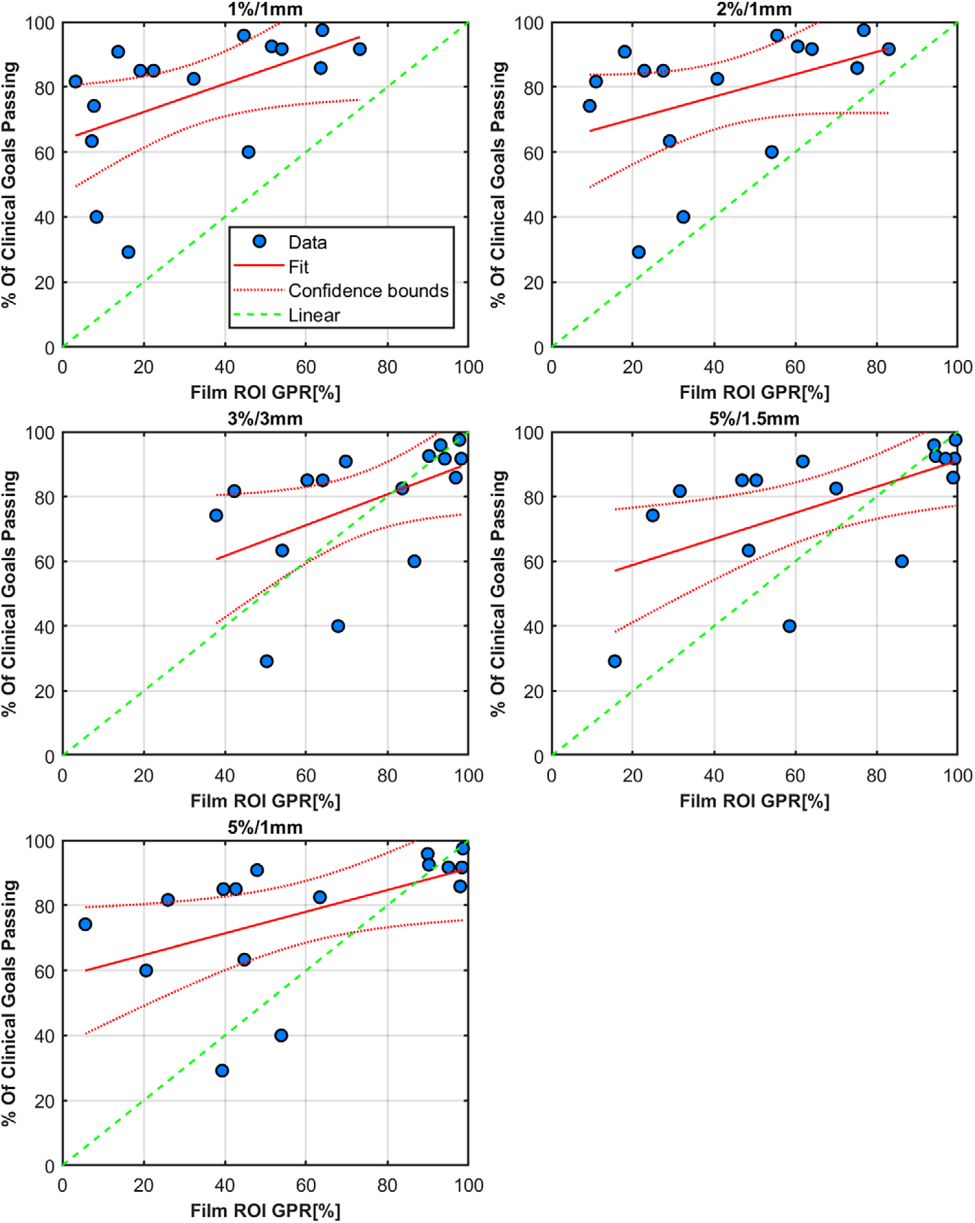
Radiochromic film results. Higher uncertainty for this case relative to the other detectors can be seen.

Radiochromic film showed the largest variation between individual measurements and the least correlation to the decrease in clinical goals passing. However, from an error detection standpoint, it is appropriate to use 5%/1 mm or 5%/1.5 mm. The extra uncertainty in film dosimetry is both a weakness and a strength in this respect. Any error is likely to be detected but the degree to which the decrease in γ pass rate is correlated to the clinical consequence is not clear. These measurements were repeated twice, once with EBT3 and another repeat on a separate machine to confirm these results. No significant changes to the results presented here were found. The film results for this work represent an outlier in terms of the hypothesis and further work is needed to investigate whether that is due to this case alone, as our clinical experience with the use of film in the context of SIMT is that it is an accurate and reproducible dosimeter when strict protocols are adhered to. We speculate that the resolution and sensitivity of the film measurement method is such that it is more sensitive than other detectors and therefore errors affect the results in unpredictable ways. More measurement cases of different plans and error‐laden plans are needed to substantiate this claim though. Figure 11 and Table 5 shows the results for radiochromic film and assessing a ROI encompassing the lesions.

**TABLE 5:** Radiochromic film GPR results for all plans measured.

Plan	Modification	1%/1 mm [%]	2%/1 mm [%]	3%/3 mm [%]	5%/1.5 mm [%]	5%/1 mm [%]	Clinical goals passing [%]
Brain 0	Original plan	64.0	76.9	97.7	99.5	98.6	97.5
Brain 1	1% scaling of MU	44.7	55.5	93.1	94.2	89.9	95.8
Brain 2	2% scaling of MU	51.5	60.6	90.3	94.6	90.2	92.5
Brain 3	3% scaling of MU	73.2	83.0	98.2	99.2	98.4	91.7
Brain 4	0.01–0.1 mm random offset	54.0	64.1	94.1	97.0	95.0	91.7
Brain 5	0.1 mm systematic	13.7	18.0	69.8	61.9	47.9	90.8
Brain 6	0.1‐0.25 mm random	63.6	75.2	96.8	98.9	97.9	85.8
Brain 7	7% scaling of MU	22.5	27.5	64.1	50.4	42.7	85.0
Brain 8	1‐degree collimator rotation	19.2	22.8	60.3	46.9	39.6	85.0
Brain 9	0.25 mm systematic offset	32.3	40.8	83.7	70.1	63.4	82.5
Brain 10	0.25–0.5 mm random offset	3.3	10.9	42.3	31.6	26.0	81.7
Brain 11	2‐degree collimator rotation	7.8	9.4	37.9	24.9	5.6	74.2
Brain 12	0.1–1 mm random offset	7.3	29.1	54.1	48.5	44.8	63.3
Brain 13	0.5 mm systematic offset	45.8	54.2	86.7	86.2	20.6	60.0
Brain 14	1 mm systematic offset	8.4	32.5	67.9	58.6	53.9	40.0
Brain 15	1–2 mm random offset	16.3	21.5	50.3	15.6	39.3	29.2

Shaded regions show optimal criteria as determined by the linear model.

### 3.5 TrueBeam log files

**FIGURE 12:**
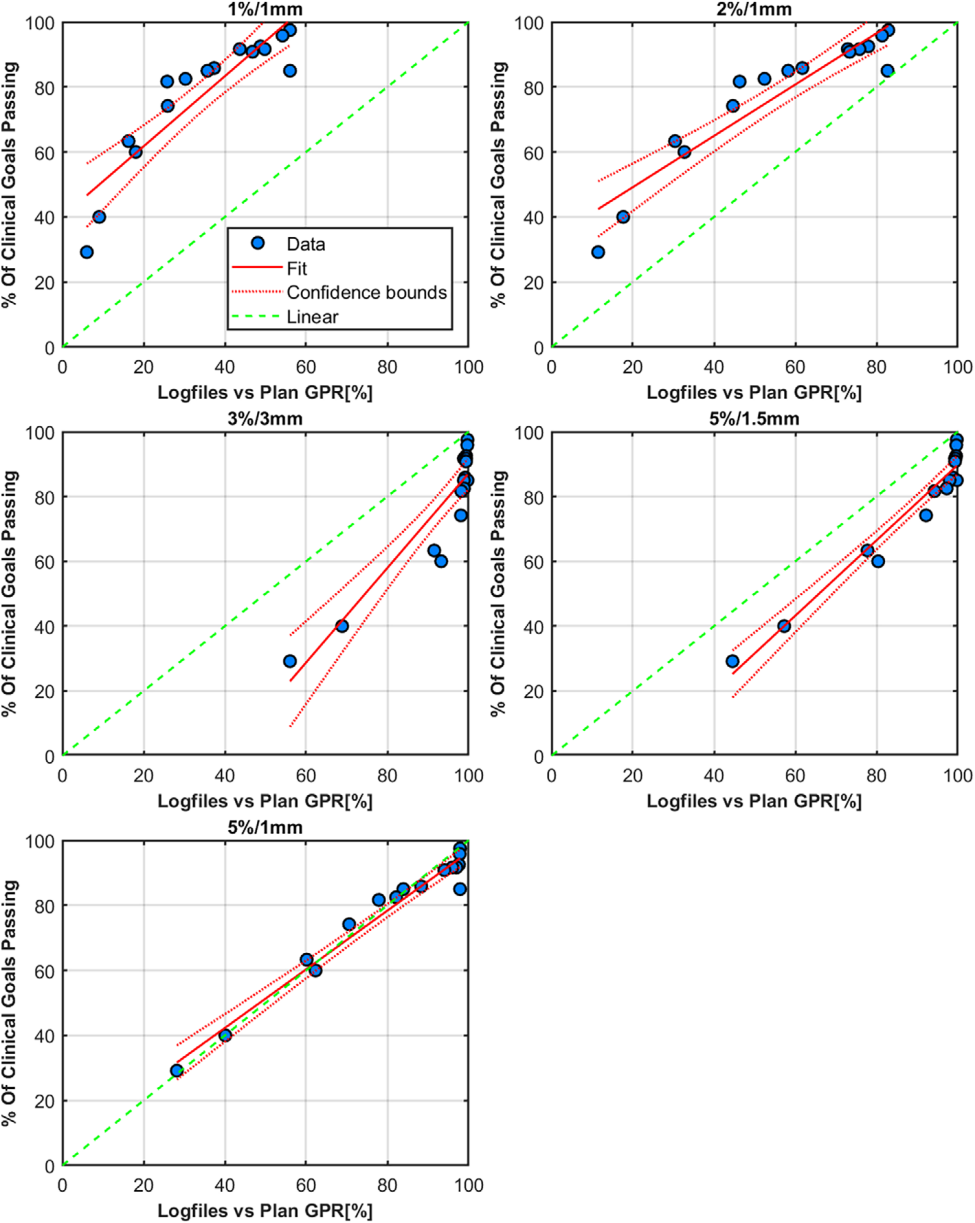
Results from TrueBeam trajectory log files compared to the treatment plan generated fluence. Each figure's title shows the γ criteria used for the analysis. Each data point is a comparison of the composite fluence (intensity map) from four fields for the plans in Table 1. Each measurement of the original and erroneous plan is compared to the original plan's composite fluence image.

**FIGURE 13:**
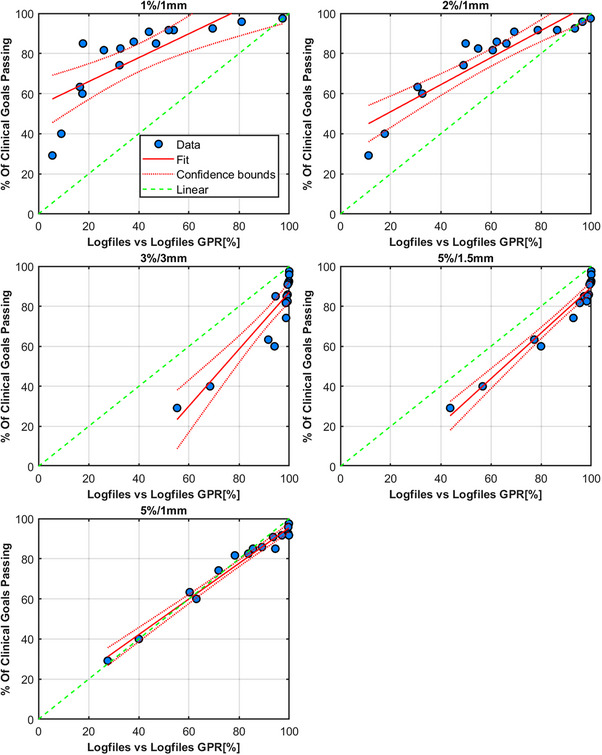
Results from TrueBeam trajectory log files. Each figure's title shows the γ criteria used for the analysis. Each data point is a comparison of the composite fluence (intensity map) from four fields for the plans in Table 1. The actual fluence based on the recorded MLC positions for each plan is compared to the expected fluence from the log file of the original plan.

The results for the TrueBeam trajectory log file method are shown in Figures 12 and 13, respectively. Tables 6 and 7 show these results in full. There are two appropriate methods for using log files. One method is to compare the actual reconstructed fluence map delivered from the machine to the reconstructed fluence map generated from the treatment plan DICOM (Figure 12) and the second method is to solely use the log file to reconstruct two sets of fluence (expected and actual delivered fluence as recorded by the linac). A fluence map that contains all the planned MLC positions (“expected”) can be compared to the measured fluence map generated from the actual MLC positions (“actual”) as well as all other mechanical axes information (Figure 13). The two methods differ in the temporal resolution of the reconstruction. Figures 12 and 13, Tables 6 and 7 show that 5%/1 mm for the log files is the optimum criteria across the range analyzed herein. Table 2 also highlights a strong correlation between the γ pass rates and the decline in the percentage of clinical goals passing for both methods.

**TABLE 6:** GPR results for all plans measured using log files recorded actual fluence reconstruction compared to the planned fluence from the treatment plan.

Plan	Modification	1%/1 mm [%]	2%/1 mm [%]	3%/3 mm [%]	5%/1.5 mm [%]	5%/1 mm [%]	Clinical goals passing [%]
Brain 0	Original plan	56.0	82.9	99.7	99.7	97.9	97.5
Brain 1	1% scaling of MU	54.2	81.4	99.7	99.6	97.8	95.8
Brain 2	2% scaling of MU	48.8	78.0	99.4	99.6	97.6	92.5
Brain 3	3% scaling of MU	43.7	72.9	98.8	99.3	97.0	91.7
Brain 4	0.01–0.1 mm random offset	49.8	75.8	99.4	99.4	96.0	91.7
Brain 5	0.1 mm systematic	46.8	73.4	99.4	99.3	94.0	90.8
Brain 6	0.1–‐0.25 mm random	37.4	61.7	99.1	98.8	88.3	85.8
Brain 7	7% scaling of MU	56.0	82.7	99.7	99.7	97.9	85.0
Brain 8	1‐degree collimator rotation	35.7	58.3	98.8	98.0	83.9	85.0
Brain 9	0.25 mm systematic offset	30.3	52.4	98.9	97.3	82.2	82.5
Brain 10	0.25–0.5 mm random offset	25.8	46.3	98.2	94.3	77.9	81.7
Brain 11	2‐degree collimator rotation	25.9	44.7	98.1	92.2	70.6	74.2
Brain 12	0.1–1 mm random offset	16.3	30.4	91.5	77.8	60.1	63.3
Brain 13	0.5 mm systematic offset	18.1	32.7	93.3	80.4	62.3	60.0
Brain 14	1 mm systematic offset	9.1	17.6	68.9	57.3	40.1	40.0
Brain 15	1–2 mm random offset	6.0	11.5	56.0	44.5	28.2	29.2

**TABLE 7:** Gamma pass rates for the log file recorded actual fluence reconstruction compared to the log file recorded expected fluence.

Plan	Modification	1%/1 mm [%]	2%/1 mm [%]	3%/3 mm [%]	5%/1.5 mm [%]	5%/1 mm [%]	Clinical goals passing [%]
Brain 0	Original plan	97.3	99.7	100.0	100.0	99.9	97.5
Brain 1	1% scaling of MU	81.0	96.5	100.0	100.0	99.6	95.8
Brain 2	2% scaling of MU	69.5	93.5	99.9	100.0	99.6	92.5
Brain 3	3% scaling of MU	53.9	86.4	99.7	99.9	99.9	91.7
Brain 4	0.01–0.1 mm random offset	51.9	78.7	99.6	99.6	97.1	91.7
Brain 5	0.1 mm systematic	44.1	69.3	99.4	99.3	93.6	90.8
Brain 6	0.1–0.25 mm random	38.0	62.3	99.3	99.0	89.1	85.8
Brain 7	7% scaling of MU	17.7	49.9	94.5	97.2	94.5	85.0
Brain 8	1‐degree collimator rotation	46.9	66.2	99.0	98.6	85.5	85.0
Brain 9	0.25 mm systematic offset	32.7	54.9	99.3	98.2	83.7	82.5
Brain 10	0.25–0.5 mm random offset	26.1	60.7	98.7	95.4	78.3	81.7
Brain 11	2‐degree collimator rotation	32.3	49.1	98.7	92.9	71.8	74.2
Brain 12	0.1–1 mm random offset	16.5	30.8	91.6	77.3	60.3	63.3
Brain 13	0.5 mm systematic offset	17.5	32.6	94.1	80.0	63.0	60.0
Brain 14	1 mm systematic offset	9.1	17.6	68.4	56.7	40.0	40.0
Brain 15	1–2 mm random offset	5.6	11.2	55.3	43.8	27.6	29.2

### 3.6 SRS MapCHECK

**FIGURE 14:**
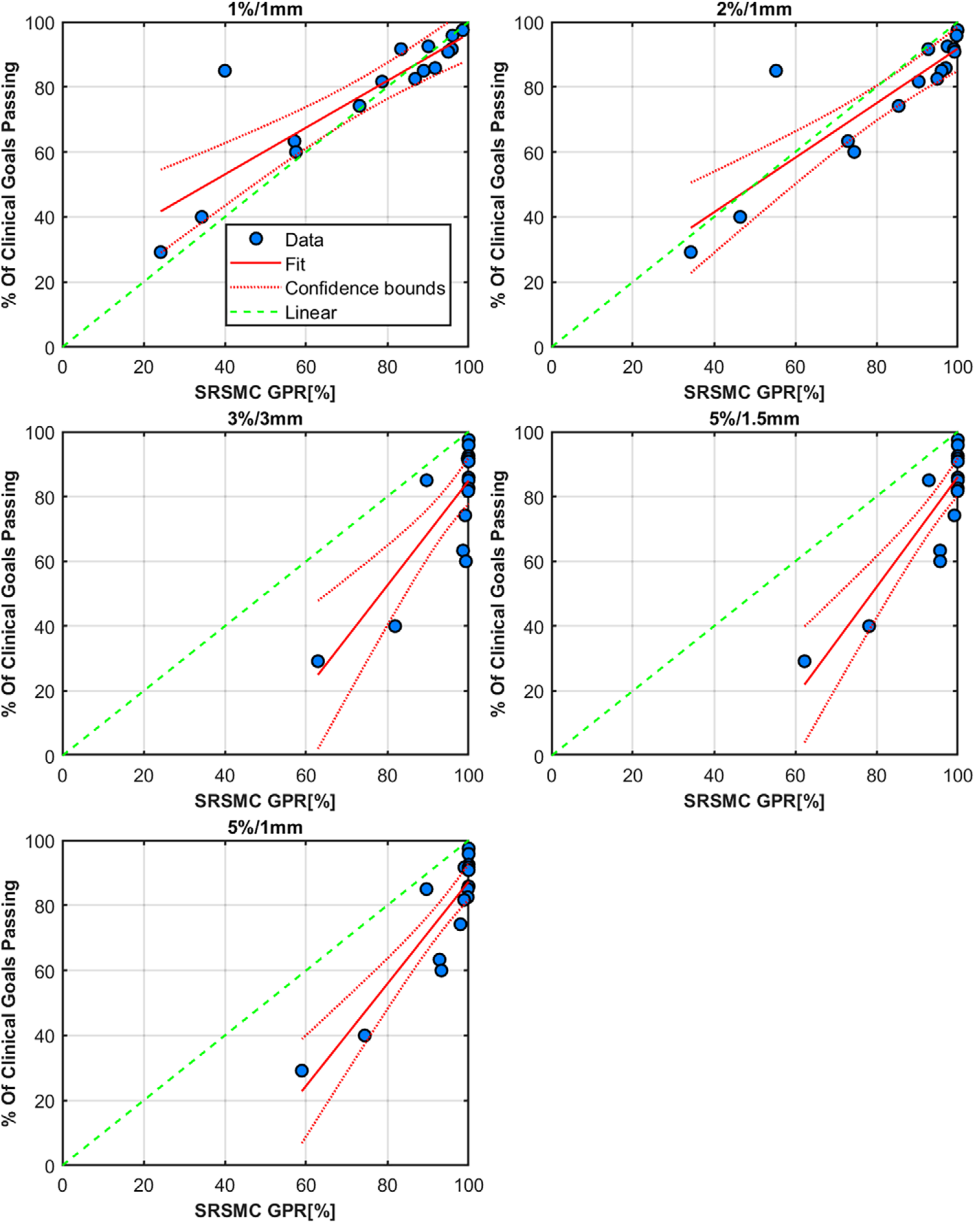
Results from Sun Nuclear's SRS MapCHECK. Each figure's title shows the γ criteria used for the analysis. Each data point is a comparison of the composite dose from four fields analyzed in SNC Patient for each plan in Table 1. Each measurement of the original and erroneous plan is compared to the original plan's dose distribution.

Results for the SRS MapCHECK measurements are shown in Figure 14 and Table 8, respectively. According to these results, the SRS MapCHECK should be used with a γ criterion of 2%/1 mm or tighter. Again, it should be noted that other criteria like 3%/1 mm or 2%/2 mm might be appropriate also, but these have not been evaluated against these plans. This device, along with all others presented in this work should not be used with loose tolerances like 3%/3 mm, which relegates any error detection ability to all but the most serious errors (Brain 14 and Brain 15 plans in this work).

**TABLE 8:** Gamma pass rates for SRS MapCHECK measurements.

Plan	Modification	1%/1 mm [%]	2%/1 mm [%]	3%/3 mm [%]	5%/1.5 mm [%]	5%/1 mm [%]	Clinical goals passing [%]
Brain 0	Original plan	98.6	99.9	100.0	100.0	100.0	97.5
Brain 1	1% scaling of MU	96.0	99.7	100.0	100.0	100.0	95.1
Brain 2	2% scaling of MU	90.1	97.5	100.0	100.0	100.0	92.5
Brain 3	3% scaling of MU	83.4	92.7	99.8	100.0	99.0	91.7
Brain 4	0.01–0.1 mm random offset	95.9	99.0	100.0	100.0	100.0	91.7
Brain 5	0.1 mm systematic	95.0	99.2	100.0	100.0	100.0	90.8
Brain 6	0.1–‐0.25 mm random	91.8	97.0	100.0	100.0	100.0	85.8
Brain 7	7% scaling of MU	40.0	55.3	89.7	92.9	89.6	85.0
Brain 8	1‐degree collimator rotation	88.9	96.0	100.0	100.0	99.8	85.0
Brain 9	0.25 mm systematic offset	86.8	94.9	100.0	100.0	99.8	82.5
Brain 10	0.25–0.5 mm random offset	78.7	90.4	99.9	99.9	98.9	81.7
Brain 11	2‐degree collimator rotation	73.2	85.4	99.2	99.2	98.0	74.2
Brain 12	0.1–1 mm random offset	57.1	73.0	98.6	95.6	92.8	63.3
Brain 13	0.5 mm systematic offset	57.5	74.5	99.3	95.7	93.3	60.0
Brain 14	1 mm systematic offset	34.3	46.4	81.9	78.2	74.4	40.0
Brain 15	1–2 mm random offset	24.2	34.3	62.9	62.3	58.9	29.2

## 4 DISCUSSION

Recommendations of AAPM Task Group No. 218 in determining tolerance limits and methodologies for IMRT‐based verification QA. In the last sentence of Section 9 it is recommended that “…efforts should be focused on further improving the correlation between IMRT QA evaluation metrics and underlying planning or delivery errors.”^22^ This work aimed to evaluate five methods for quality assurance of SIMT treatment plans according to the methods’ suitability and sensitivity to delivery errors using a novel correlation between γ pass‐rates and the clinical plan quality degradation due to the error. We introduced a novel method to determine optimal γ criteria for each method, correlating error severity with its detection based on its impact on clinical plan integrity, as measured by the decrease in clinical goal achievement compared to the original plan. This method can be used to establish appropriate gamma criteria by correlating gamma pass rates at a particular dose/dta criterion to clinical plan degradation.

Errors introduced into the original plan, along with their effect on clinical goals are given in Table 1. The key findings of this work were that this novel method can be applied to an assessment of any detector for PSQA use, and provides a way to determine the optimal γ criteria for the detector, to maximize the detector's error detection capability. A second key finding was that loose γ criteria for PSQA, for example, 3%/3 mm coupled with the detector choice and its use‐case applicability, can result in clinically relevant false positives, where a plan that should fail QA and detect a serious, clinically relevant delivery issue, passes the test. This was found across all detectors and methods presented in this work, except for radiochromic film and we recommend that these loose criteria be tightened to maximize error detection. All detectors and methods studied in this work demonstrate that errors can be detected reliably, provided that the appropriate γ criteria are used.

For the ArcCHECK, a criterion of 2%/1 mm should be investigated for a range of patient cases experimenting with looser criteria like 2%/2 mm and 3%/2 mm given the resolution of the device, in line with recommendations from AAPM TG‐218.^22^ ArcCHECK measurements of the plans can be complemented by an evaluation of the couch walk‐out and IGRT procedures as the device is only able to be used without couch rotation. The same applies to the use of PD and log file‐based methods, where no information about the spatial accuracy of the dose delivery is obtained in a phantom. Whilst this work provides a starting point for appropriate tolerance selection in the context of SIMT, it is recommended that each facility investigate its own appropriate criteria, as individual clinical cases may necessitate looser or tighter tolerances depending on the site.

SIMT cases are among the most complex radiotherapy plans yet there is no clear guidance on which detectors and/or γ criteria should be used when performing PSQA. AAPM TG‐218^22^ recommends universal *tolerance limits* where the γ passing rate should be ≥ 95%, with 3%/2 mm and a 10% dose threshold, and universal *action limits* where the γ passing rate should be ≥ 90%, with 3%/2 mm and a 10% dose threshold. These limits serve as a good starting point for PSQA of IMRT and VMAT treatment plans. With SIMT, tighter tolerances depending on the equipment available should be investigated, such as 2%/1 mm to detect subtle regional errors and to discern if the errors are systematic for a specific treatment site or delivery machine. The reduction to 1 mm distance‐to‐agreement is also recommended regardless of dose‐difference criteria given the tighter margins often employed in SIMT treatment plans.

This work echoes the findings of Xia et al.^23^ In their work, the authors reported on their experience with applying TG‐218 recommendations to a large multicenter clinical SRS and SBRT program for a range of diverse clinical pre‐treatment QA systems. Pre‐treatment QA systems included Delta4 (Scandidos), PD, ArcCHECK, and SRS MapCHECK, three of which were also evaluated in our work. Their work found that by applying TG‐218, a 3%/1 mm criterion for SRS cases and 4%/1 mm for SBRT cases can be applied while still maintaining an in‐control QA process. These criteria are in line with what we have recommended in this work based on our modeling. Their work showed that applying the TG‐218 recommendations to SRS and SBRT cases resulted in more stringent gamma criteria with a higher action level than the generalized passing rate for all devices in the study. The study found that compared to the standard criteria of 3%/3 mm, tighter criteria of 3%/1 mm for SRS and 4%1 mm for SBRT cases using Delta4 and PD, 3%/2 mm for ArcCHECK and 3%/1 mm for SRS MapCHECK SRS cases could be applied with acceptable action and tolerance limits. In agreement with this work, it was shown that stringent criteria (2%/1 mm) could be applied for multiple target SRS using the SRS MapCHECK.

James et al.^24^ compared commercial quality assurance (QA) devices (EBT‐XD film, IBA Matrixx Resolution, SNC ArcCHECK, Varian aS1200 EPID, SNC SRS MapCHECK, and IBA myQA SRS) to film dosimetry for pre‐treatment evaluation of stereotactic radiosurgery (SRS), fractionated SRT, and stereotactic body radiation therapy treatment plans. Their work compared gamma pass rates for a set of forty plans as well as two plans containing MLC positioning error scenarios. Their work found that errors in MLC positioning were most reliably detected at 2%/1 mm for high‐resolution detectors and that lower‐resolution detectors did not consistently detect MLC positioning errors. Our work also confirms their findings with 2%/1 mm being the most appropriate for the SRS MapCHECK and Portal Dosimetry. Our findings differ concerning the ArcCHECK where their findings suggest that this detector, on average, did not correctly identify the changes in the dose distribution when lagging MLC error plans were measured. This could be due to the nature of the error introduced compared with this study and the plan's complexity.

There are several limitations to our work and areas where the work can be expanded. This work is based on the results of one patient plan that was subsequently modified and measured on a range of devices. Future work aims to reduce the number of plans in Table 1 and test this method across a wide range of treatment plans and this would overcome one of the shortcomings of this study, where plan variation was not a variable that was studied. Future work internally at our organization aims to use this method to determine treatment plan robustness to these effects across a large patient group.

Whilst this work provides recommendations on dose/spatial gamma criteria for these detectors, it is important to understand the limitations of each detector and methodology (see Appendix: Table A1) and to establish center and site‐specific tolerances according to TG‐218 methodology where possible. It is important to note that although TG‐218 does not specifically address the topic of stereotactic radiotherapy, its methodological principles can be applied to the establishment of best‐practice gamma criteria and tolerances for each organization and detector. Further, all initial gamma criteria should be tightened/refined where applicable based on data acquired for a range of patient cases over time. In this work, we have demonstrated that all detectors and methods outlined herein can be used to detect clinically relevant errors on a TrueBeam linear accelerator.

This work also shows the potential usefulness of a combinatorial approach for QA of these cases. For example, rather than processing 20 film measurements, one per PTV, the entire delivery might be captured on an ArcCHECK with no couch rotation, to determine the composite deliverability, and then a single film plane measurement done to account for the shortcomings of the ArcCHECK method and focus in on the agreement in areas of steep‐dose gradient, while assessing the couch‐walkout and IGRT workflow. This approach coupled with a 3D independent plan recalculation provides a robust way to ensure the planning system and delivery errors do not affect treatment efficacy and combinatorial QA may reduce the risk of adverse events.

Though gamma criteria are tightened for ArcCHECK, SRS MapCHECK and Portal dosimetry, the results discussed show an acceptable pass rate of > 95%. Therefore, we suggest that if using clinically, the standard tolerance of > 95% gamma pass rate be considered. In the case of pass rates falling below the 95% threshold, the standard criteria of 3%/1 mm be applied to evaluate the results and could be further confirmed by assessing the log files collected from the QA delivery. However, this would highly depend on the department's practice. Retrospective studies with tighter criteria applied may be a starting point prior to clinical application.

## 5 CONCLUSION

SIMT plans, though optimized to deliver highly conformal dose distribution to multiple volumes with acceptable toxicity, require a safe and efficient method of validation for delivery. As the number of volumes targeted in a single field increase, the complexity and time required for patient specific QA increases. In this work, we aimed to assess five methods for quality assurance of SIMT treatment plans in terms of their suitability and sensitivity to delivery errors and machine miscalibration. We also proposed a novel method for setting appropriate gamma criteria for each device and demonstrated the following: 2%/1 mm is a good starting point for the ArcCHECK, PD, and the SRS MapCHECK methods respectively, and provides clinically relevant error detection sensitivity. Looser gamma criteria of 5%/1 mm or 5%/1.5 mm are suitable for film dosimetry and log‐file‐based methods. From these starting points, we recommend evaluating SIMT patient‐specific QA results against a cohort of representative patients with a range of PTV sizes, quantities, and distances from the isocenter. The tighter criteria for the devices other than the film and log‐file‐based methods, may result in pass rates that lie on the threshold of pass/fail criteria. At these times, dose‐volume‐based criteria to PTV and OAR may play a role in deciding the acceptance of borderline pass rates. Further work is required to correlate the results to dose‐volume‐based criteria. Combinatorial approaches using multiple detectors may also be used to mitigate each detector's drawbacks and enhance the robustness of the case evaluation.

## AUTHOR CONTRIBUTIONS

L. Dunn conceived the presented work and developed the theory, as well as performed the programming and acquired the measurements in conjunction with the listed co‐authors. T. Te Ruruku, A. Tamborriello, B. Subramanian, and X. Xu performed measurements and helped analyze the findings of this work. All authors discussed the results and contributed to the final manuscript.

## CONFLICT OF INTEREST STATEMENT

The authors declare no conflicts of interest.

## Appendix

**TABLE A1:** Summary of the detectors used in this study and a summary list of advantages and disadvantages in the context of stereotactic radiotherapy patient‐specific QA.

Detector	Advantages	Disadvantages
Sun Nuclear ArcCHECK (TC, minus couch)	Total array diameter of 21 cm capturing the entire field. Can perform angular corrections. Accurate and reproducible Well‐established in clinical practice. Can be used on larger field (> 4 cm) non‐coplanar stereotactic patients.	Cannot be used for non‐coplanar fields. 1386 diodes. Detector spacing of 1 cm, poor spatial resolution for smaller field stereotactic measurement conditions. Low spatial resolution
Varian PD (PC)	An active area of 40 cm x 30 cm for the Image Detector Unit (IDU) and 43 cm x 43 cm for the Digital Megavoltage Imager (DMI) Does not require a phantom or setup time. Well established on the Varian platform.	Low spatial resolution compared to film. Not independent of the linac or manufacturer as it is attached to the gantry (can't measure gantry sag) A high measurement workload potentially reduces the life of the EPID panel. Not tissue equivalent. Over‐response to low‐energy MLC transmission.
Radiochromic Film (TC)	Excellent spatial resolution Able to accurately capture high‐dose gradients. Energy independent Angle independent Dose rate independent Tissue equivalent Can be used for non‐coplanar fields. Can be used to capture most PTVs in a single measurement (Utilizing CIRS Multi‐Lesion Brain QA phantom).	Cost per box (consumable material). Price changes depending on the type chosen and dose level EBT3 vs. EBT‐XD Time‐consuming in proportion to the number of PTVs Post‐processing time delay (can be mitigated with multiple calibration curves) Accuracy and reproducibility are highly process‐ and user‐dependent.
Log files (NA)	High‐frequency (20 ms/50 Hz) real‐time data from the Linac about the actual and planned positions of each mechanical axes. Contains information about the gantry, couch, collimator, MLC, and MU delivered. No detector is required. Can be used to generate high‐fidelity fluence maps. Processing can be automated to obtain per‐fraction verification. Detects transfer errors when compared against plan. Can be easily combined with other measurements for a more robust verification.	In isolation, they are a surrogate for an actual measurement. Do not consider physical phenomena like gantry sag, couch walkout, collimator walkout, and gantry MV isocenter congruence and size. No imaging component Beam characteristics and interplay with the mechanical collimator information were not obtained.
Sun Nuclear SRS MapCHECK (TC)	1013 diodes 0.48 mm^2^ active detector area 2.47 mm detector spacing Can be used for non‐coplanar fields	Detector area 77 × 77mm^2^ – cannot be used to measure large targets or capture multiple targets far apart. As the number of PTVs becomes > 3, measurement shifts can become cumbersome relative to a single capture delivery with film. The manufacturer produces a separate phantom at an additional cost (which can be mitigated with in‐house designs). Cost

“(TC)” indicates True Composite, “(PC)” indicates perpendicular composite according to AAPM TG‐218.
